# Associations between anthropometry, body composition, and body image in athletes: a systematic review

**DOI:** 10.3389/fpsyg.2024.1372331

**Published:** 2024-05-13

**Authors:** Mary D. Webb, Melissa M. Melough, Carrie P. Earthman, Sarah E. Katz, Carly R. Pacanowski

**Affiliations:** ^1^Department of Health Behavior and Nutrition Sciences, College of Health Sciences, University of Delaware, Newark, DE, United States; ^2^Research and Engagement Department, Library, Museums, and Press, University of Delaware, Newark, DE, United States

**Keywords:** anthropometry, body composition, body image, athletes, sport

## Abstract

**Introduction:**

Poor body image is a potent risk factor for disordered eating and eating disorders. Athletes are a population at increased risk for eating disorders despite reports of lower body image concerns compared to non-athletes. Body size and composition may influence an athlete’s susceptibility to poor body image.

**Methods:**

Five electronic databases (PubMed, Cochrane Library, PsycINFO, Web of Science, SPORTDiscus) were searched to systematically evaluate the literature regarding the association between body measures (i.e., anthropometric and body composition indicators) and body image in athletes. The systematic review was completed following PRISMA guidelines and 27 cross-sectional studies were identified for inclusion and evaluated using the Joanna Briggs Institute Critical Appraisal Checklist for Analytical Cross-Sectional Studies.

**Results:**

Studies differed in methodological assessment of anthropometry or body composition (i.e., self-reported versus researcher-measured), methods for evaluating aspects of body image, geographic location, and sport type. Higher body mass index (BMI) or percent body fat (%BF) was significantly associated with greater body dissatisfaction in 16 of 22 studies (72.7%). Positive associations between body measures and aspects of negative body image were most consistently observed among studies that assessed BMI based on self-reported heights and weights, while significant associations between body composition measures (e.g., %BF, fat mass, fat-free mass) were less common. Four of seven studies assessing relationships between BMI and an aspect of positive body image reported significant inverse relationships, while three revealed insignificant associations.

**Discussion:**

Overall, higher BMI and body fat were associated with body dissatisfaction among athletes. Future studies are needed to confirm these findings within focused populations and utilizing body composition methods (e.g., bioelectrical impedance techniques).

**Systematic review registration:**

https://www.crd.york.ac.uk/prospero/, CRD42023446518.

## 1 Introduction

Body image is a multidimensional concept that includes perceptual, cognitive, affective, and behavioral facets (Wertheim and Paxton, 2011). The negative and positive dimensions of body image are two distinct constructs (Tylka and Wood-Barcalow, 2015b), such that individuals can experience aspects of negative and positive body image simultaneously (Tylka, 2011; Tylka and Wood-Barcalow, 2015b). However, negative body image is an established risk factor of disordered eating and eating disorders, while positive body image is recognized as a protective factor against eating disorder symptoms (Stice and Shaw, 2002; Piran, 2015; Glashouwer et al., 2019; Burychka et al., 2021; Linardon, 2021). Negative body image includes body image disturbance (BID), which can manifest as perceptual (e.g., body image distortion) or attitudinal disturbances (e.g., body dissatisfaction) (Gardner, 2011), while positive body image includes characteristics such as body appreciation and acceptance (Tylka and Wood-Barcalow, 2015b). BID has been consistently implicated in the etiology, symptomatology, and maintenance of disordered eating and eating disorders, in general populations (Stice, 2001; Stice and Shaw, 2002; Glashouwer et al., 2019) and in samples of athletes (Kong and Harris, 2015; Karrer et al., 2020).

Athletes are at increased risk for disordered eating and eating disorders (Stice and Shaw, 2002; Piran, 2015; Linardon, 2021). Specific athlete subpopulations (e.g., gymnasts, figure skaters) have been found to be at higher risk for disordered eating (Petrie and Greenleaf, 2011; Berengüí et al., 2023) due to general societal pressures and additional sport environment pressures including judging criteria (e.g., scoring based on appearance in sports like diving, cheerleading, gymnastics), uniform requirements, weight requirements, and performance demands (Petrie and Greenleaf, 2007; Petrie, 2020). Disordered eating and eating disorders can be detrimental to athletic performance, as clinical characteristics of anorexia (e.g., low body weight) and bulimia nervosa (e.g., purging) may increase injury susceptibility and negatively affect sport performance (El Ghoch et al., 2013). Individuals with longstanding eating disorders have demonstrated lower muscle strength and aerobic fitness (El Ghoch et al., 2013). Therefore, given the high prevalence of disordered eating and eating disorders among athletes (0–19% in males; 6–45% in females) (Bratland-Sanda and Sundgot-Borgen, 2013) and the association between negative body image and disordered eating and eating disorder risk (Stice and Shaw, 2002; Glashouwer et al., 2019), it is critical to understand additional factors affecting body image within athlete populations.

In the present review, body measures include anthropometric indicators like skinfold thickness and body mass index (BMI), as well as body composition indicators [e.g., percent body fat (%BF), fat mass (FM) and fat-free mass (FFM)] (Table 1). The body composition indicators may be estimated with techniques like bioelectrical impedance analysis (BIA) and dual-energy x-ray absorptiometry (DXA), or with more simple methods such as applying established equations that utilize the sum of multi-site skinfold thickness measurements (Jackson and Pollock, 1978; Jackson et al., 1980; Slaughter et al., 1988). In the present review, the anthropometric indicators of interest were those commonly associated with or used to approximate various aspects of body composition (e.g., BMI, regional skinfolds, regional circumferences) (Table 1). BMI is an example of a body measure that has been well-documented as a physical factor that is associated with body image in adolescents and adults (Paxton et al., 2006; Calzo et al., 2012; Bucchianeri et al., 2013; Weinberger et al., 2017). Higher BMI has shown positive associations with components of negative body image (e.g., body dissatisfaction) (Paxton et al., 2006; Calzo et al., 2012; Bucchianeri et al., 2013; Weinberger et al., 2017) and inverse associations with aspects of positive body image (e.g., body appreciation) (He et al., 2020). However, the research to date has primarily focused on non-athlete populations. Given that athletes are exposed to sport-specific constraints (e.g., weigh-ins) and judgments (e.g., performance scoring), we seek to understand whether this link between body measures and body image exists in athletes as well.

**Table 1:** Body measures assessed in the present review, classified as anthropometric or body composition indicators.

Anthropometric		Body composition	
Indicator	Method	Indicator	Method(s)
Body mass index (BMI)	Height and weight (self-reported or measured)	Percent body fat (%BF)	Application of multi-site skinfold measurements to equations
Regional skinfold thickness	Calipers	Fat mass (FM)	Dual-energy x-ray absorptiometry (DXA)
Regional circumference	Measuring tape	Fat-free mass (FFM)	Bioelectrical impedance analysis (BIA) or spectroscopy (BIS)
		Lean body mass (LBM)	
		Fat-free mass index (FFMI)	

Most studies and reviews have focused on associations between BMI, a crude proxy of body fat, and body image, rather than evaluating more accurate indicators like %BF or FFM. Exploring the relationship between anthropometric and body composition indicators, and body image may provide more nuance to the current state of knowledge and disentangle the distinct influences of different body composition components among athletes. BMI does not provide complete information on the contribution of the various body compartments (e.g., FM and FFM) to body weight (Kyle et al., 2003), and may be less useful in athletic populations with greater muscle mass (Lukaski and Raymond-Pope, 2021). Given the limitations of BMI, it is plausible that BMI and body composition measures may be differentially related to body image. For example, among athletes, BMI and body fat percentage may be positively associated with negative body image, while an opposite relationship may exist with FFM. The sex and sport-related differences in body composition further underscore the importance of exploring associations of body components and body image (Bredella, 2017; Lukaski and Raymond-Pope, 2021). The present review will critically examine the associations between anthropometric indicators and body image and compare those to associations of body composition indicators and body image.

The relationships between anthropometry, body composition, and body image in athletes have been evaluated in individual studies and briefly as sub-aims of 2001 (Hausenblas and Symons Downs, 2001) and 2023 systematic reviews and meta-analyses (Zaccagni and Gualdi-Russo, 2023). However, this recent review did not prioritize body composition in the search strategy or analyses, did not assess any elements of positive body image, and did not specify or adhere to a consistent definition of athletes. Identifying and understanding factors related to athlete body image will allow future research efforts to focus on specific populations to prevent or combat disordered eating behaviors. Therefore, this review aims to systematically assess the literature to explore the relationship between anthropometry, body composition, and aspects of negative and positive body image among athletes.

## 2 Materials and methods

This systematic review was guided by the Preferred Reporting Items for Systematic Reviews and Meta-Analyses (PRISMA) guidelines (Page et al., 2021). The PRISMA checklist can be found in the supplementary materials (Supplementary Table S1). The review was registered in the international prospective register of systematic reviews, PROSPERO, registration number CRD42023446518.

### 2.1 Search strategy

The search strategy was developed in consultation with a research librarian (SEK). The search was conducted in July 2023 using the following electronic databases: PubMed, Cochrane Library, APA PsycINFO (ProQuest), Web of Science, and SPORTDiscus with Full Text. The search was developed in PubMed and modified for each database. Keywords and controlled vocabulary (e.g., PubMed’s Medical Subject Headings and PsycINFO’s Thesaurus) were combined by Boolean logic (AND, OR) as appropriate in each database to return relevant records. Date boundaries were applied on the initial database searches. Search terms related to population included the keywords ‘athlete,’ ‘sport,’ and ‘player,’ as well as terms related to sports in which athletes are not referred to as players (e.g., dancing, running, etc.). Terms for anthropometric and body composition measurements were determined in consultation with an expert in the field of body composition (CPE) and derived from prior systematic reviews investigating body composition in athletes (Campa et al., 2021; Sansone et al., 2022). Body image terms included those related to overall and attitudinal body image (e.g., body dissatisfaction, body satisfaction). Hand searching of the reference lists of included reports was also conducted. Supplementary Table S2 contains the complete details of search terms and search strategies for each database.

### 2.2 Inclusion and exclusion criteria

To be included in the review, studies needed to meet the following eligibility criteria: (a) published in a peer-reviewed journal; (b) primary research articles; (c) available in the English language; (d) included a sample of current athletes; (e) published between January 2011 and July 2023; (f) anthropometric or body composition indicators were reported for the athletes; (g) assessed body image with a validated tool; and (h) quantitatively assessed the relationship between anthropometric or body composition indicators and body image in athletes alone. January 2011 was selected as the start of the search range due to the rapid increase in social media usage in the late 2000s and early 2010s (Madden et al., 2013). In 2010, the image-based application Instagram was created, and this also represents the year in which over three-quarters of teens and 50% of adults reported using social networking sites (Madden et al., 2013). Given the demonstrated associations between social media usage and body dissatisfaction related in part to the portrayal of unrealistic and stereotypical beauty ideals and the notable communicative aspects of modern social media (Perloff, 2014; Vuong et al., 2021), we wanted to ensure that our search considered this important sociocultural factor. Therefore, to include research reflecting these social media usage changes, we selected 2011 as the start date.

Studies were excluded if the participants were former (i.e., not current) athletes. Studies were also ineligible if the main purpose was to validate a new body image assessment tool, as this was incompatible with the inclusion criterion (g). The full list of inclusion and exclusion criteria can be found in Table 2.

**Table 2:** Inclusion/exclusion criteria used in screening.

Inclusion	Exclusion
Primary research study	Review, conference abstract, etc.
Published in a peer-reviewed journal	Not published in a peer-reviewed journal
Published January 2011–July 2023	Published prior to 2011
Full-text available in the English language	Full-text not available in the English language
Population includes a sample of current athletes	Do not include a sample of current athletes
Anthropometric or BC data available for athletes	No anthropometric or BC data available for athletes
Assess BI with a validated tool	Do not assess BI with a validated tool or primary aim is to validate new assessment tool
Quantitatively assess the relationship between anthropometry and/or BC and BI in athletes alone	Do not quantitatively assess the relationship between anthropometry and/or BC and BI, or not separately in athletes

BC, Body Composition; BI, Body Image.

For this review, athlete status was based on a modified version of the European Society of Cardiology (ESC) definition, which defines an athlete as “an individual of young or adult age, amateur or professional, who is engaged in regular physical training and participates in official competitions” (Pelliccia et al., 2005). The ESC defines official sports competition (local, regional, national, or international) as “an organized team or individual sports event that, placing a high premium on athletic excellence and achievement, is organized and scheduled in the agenda of a recognized Athletic Association” (Pelliccia et al., 2005). Participation in official sports competitions was considered met if directly stated or based on the affiliated sports organization or competition (e.g., National Collegiate Athletics Association, World Gymnastics Championships, Special Olympics, etc.). We classified sports as lean or non-lean, when possible. In this review, lean sports include aesthetic, weight-dependent, and endurance sports, while ball game, power, and technical sports are designated non-lean (Sundgot-Borgen, 1994).

If the participation in competition or sports organization was not explicitly stated, the author was contacted to clarify the status of their study participants. In cases when contact with study authors was unsuccessful, determination was guided by the recommendations of McKinney et al. (2019) to assess the intent of the participant for classification as an athlete or exerciser (McKinney et al., 2019). Participants that could be classified as exercisers were excluded from this review. Exercisers participate in physical activity primarily to maintain health and fitness status (McKinney et al., 2019).

Body image is a multidimensional concept and this review focused on the attitudinal dimension, which encompasses the feelings an individual has about their body size and shape (Cornelissen et al., 2019). For this review, the phrase “aspects of negative body image” will be used to encompass concepts related to attitudinal body image disturbance, such as body dissatisfaction and other negative attitudes towards the body. “Aspects of positive body image” will refer to true concepts of positive body image, such as body appreciation, as well as body satisfaction scales where higher scores indicate a greater level of body satisfaction.

### 2.3 Screening

**Figure 1:**
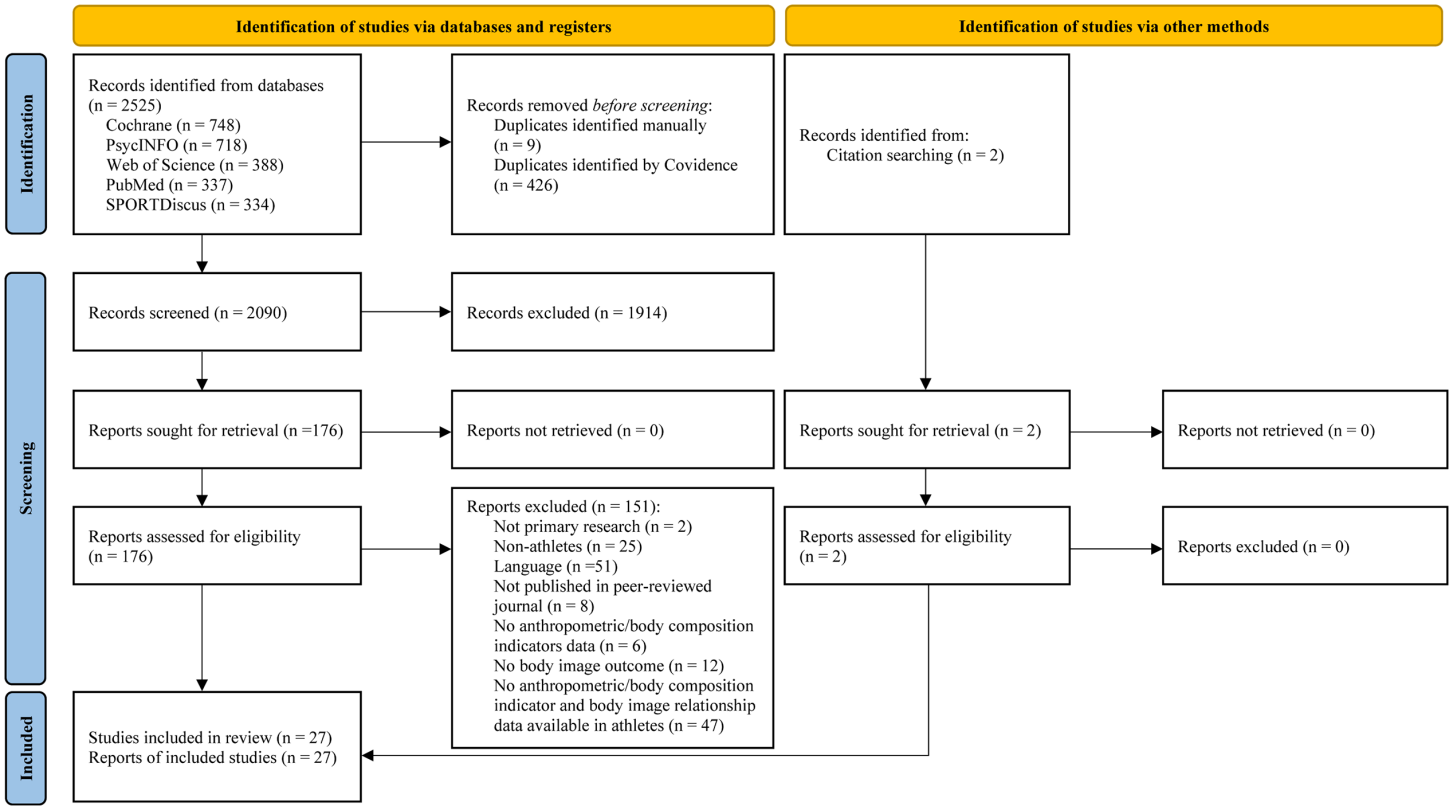
The PRISMA 2020 guidelines are referenced in the manuscript (Page 2020).

After automatic (i.e., Covidence software) and manual deduplication, titles and abstracts were screened independently by two reviewers (MDW, MMM), with decisions made based on the inclusion/exclusion criteria (Table 2). Discrepancies were resolved by a third reviewer (CRP). Reports that passed title and abstract screening were retrieved for full-text review by two independent reviewers (MDW, MMM). Title and abstract screening and full-text review were conducted with Covidence software. The full screening process for the systematic review, including the reasons for exclusion at each screening stage, is depicted in the PRISMA flow diagram (Figure 1).

### 2.4 Data extraction

Upon completion of screening, the following data were extracted into a preformatted data extraction form: author, year, country, sample size, study design, participant demographics, sport(s), competition level, anthropometric and/or body composition method(s) and indicator(s), body image assessment method(s), main body image outcome(s) relevant to this review, and quantitative relationship between anthropometric and/or body composition indicators and body image outcome(s). This step was completed by one reviewer (MDW) and cross-checked by a second independent reviewer (MMM) for accuracy and consistency.

### 2.5 Data synthesis

A narrative synthesis was used to describe the relationship between anthropometric and/or body composition indicators and body image in athletes. The strength of the associations was considered, although a meta-analysis could not be performed due to the heterogeneity of the participants and the anthropometric, body composition, and body image methods among the studies included. Tabular synthesis of the data was conducted, and studies were sub-classified according to body image outcome (i.e., negative vs. positive body image). Within each subgroup, results were further analyzed according to pertinent variables assessed in previous body image reviews (Hausenblas and Symons Downs, 2001; Burgon et al., 2023) (e.g., age, gender, sport type), as well as variables unique to the present review (e.g., anthropometric and body composition indicators).

Given that gender differences may exist in the association between BMI or body composition and body image, we assessed potential gender-related differences in this review (Murnen, 2011). Although the terms “female” and “male” designate sex, they are used throughout this review to reflect the terminology used in most of the included studies (National Academies of Sciences Engineering Medicine, 2022). The majority of studies did not indicate whether participants self-reported sex assigned at birth or gender identity. For the limited number of studies explicitly reporting gender, the terms “women” and “men” will be used to reflect the data collected.

### 2.6 Risk of bias assessment

The quality of the included studies was assessed with the Joanna Briggs Institute (JBI) Checklist for Analytical Cross-Sectional Studies (Moola et al., 2020) (Supplementary Table S3). The checklist consists of eight criteria that assess the risk of bias of the study based on the inclusion criteria, subjects and setting, validity, and reliability of the “exposure” (anthropometrics/body composition) and “outcome” (body image) measurements, assessment of confounding factors, and statistical analyses. Possible responses for each criterion are “yes,” “no,” “unclear,” or “not applicable.”

All studies were evaluated against the JBI checklist by the lead author (MDW), while a second (MMM) cross-checked the assessments for accuracy and consistency. Although JBI does not provide a total score (Moola et al., 2020), the number of “yes” responses was tallied. Studies that received >6 “yes” responses were considered to have a low risk of bias, while those with 3–5 and 0–2 “yes” scores were considered studies with a moderate and high risk of bias, respectively.

## 3 Results

A total of 2,525 records were identified from the five electronic databases searched. 2090 remained after duplicates were removed. Based on the title and abstract, an additional 1914 records were excluded. Full-text review was completed on the remaining 176 records, of which 151 were excluded and 25 met eligibility criteria. Hand-searching the reference lists of included studies identified two additional reports for inclusion (Francisco et al., 2013; de Sousa Fortes et al., 2015b), leading to a total of 27 studies included in this review (Figure 1).

### 3.1 Study characteristics

All included studies were cross-sectional. Fifteen of the studies (55.6%) were published in the last 5 years. Studies were conducted across 5 continents and 11 different countries, with the majority completed in Brazil (*n* = 9; 33.3%), the United States (*n* = 7; 25.9%), and Spain (*n* = 3; 11.1%). Individual study sample sizes ranged from 18 to 627.

Of the studies examining aspects of negative body image, most assessed general body dissatisfaction, but some investigated dissatisfaction related to specific body parts or areas, like muscle (Galli et al., 2015; de Sousa Fortes et al., 2015a; Hernández-Martínez et al., 2017). For studies using figure rating scales (FRS), the difference between perceived or current body image and ideal body image was used for this review, as the magnitude of the difference is considered a measure of body image dissatisfaction (Heider et al., 2018). Importantly, sport-related body dissatisfaction (BD-S) was also included as an outcome in three studies (Voelker et al., 2014, 2018; Aleksic-Veljkovic and Djurovic, 2020). These studies used FRS and asked participants to select the ideal figure for the given sport.

### 3.2 Risk of bias

Thirteen (48.1%) of the examined studies showed a low risk of bias, with at least 6 “Yes” responses on the JBI Critical appraisal checklist, and the remaining 14 studies (51.9%) were found to have a moderate risk of bias (3–5 “Yes” responses) (Supplementary Table S3).

In analyzing the risk of bias by body image outcome, half of the studies (*n* = 11; 50%) assessing an aspect of negative body image had a low risk of bias, while three of the seven studies (42.9%) assessing a component of positive body image were determined to have a low risk of bias.

All studies used validated tools to assess body image, as this was an inclusion criterion. Ten (37.0%) studies provided sufficiently detailed information regarding study participants and the setting. All but one study used appropriate statistical analyses. Sixty-three percent of studies (*n* = 17) identified confounding factors and used strategies to deal with these factors. According to the risk of bias assessment results, the most common criteria that studies failed to satisfy were related to providing adequate details regarding study setting and/or participants (*n* = 17; 63.0%) and utilizing an objective, standard criteria used for selecting or defining the participant population (*n* = 11; 40.7%).

### 3.3 Participants

There was heterogeneity in participant populations between and within many of the included studies. Specifically, participants differed in sex, age, sport type, and competition level. Thirteen studies (48.1%) included only females, six (22.2%) only males, and eight (29.6%) included participants of both sexes. Most athletes were 15–25 years old, but certain studies included primarily younger (Mockdece Neves et al., 2016; Kosmidou et al., 2018) (Mean (Standard Deviation (SD)): 13.3 (1.9) and 12.4 (1.7), respectively) or older (Whitehead et al., 2020; Cardoso et al., 2021) (Mean (SD): 31.4 (8.1) and 31.5 (8.6), respectively) athlete populations.

Participants competed in a variety of lean (e.g., gymnastics, figure skating, cross country, wrestling) and non-lean sports (e.g., football, basketball, soccer). Gymnastics was the most common sport. Nearly half of the studies (*n* = 12; 44.4%) included participants from multiple sports, ranging from 2 to 18 unique sports within one study. Athletes in the included studies competed at the regional, national, and international levels.

### 3.4 Body measures assessment

Fourteen of the 27 included studies (51.9%) administered self-reported questionnaires to collect participant height and weight data. Height and weight were used to calculate BMI in 25 of 27 studies (92.6%), yet 22 (81.5%) reported relationships between BMI and body image. %BF was evaluated in 11 studies (40.7%); nine (33.3%) used skinfold measurements (de Sousa Fortes et al., 2013, 2015a,b, 2020; Goltz et al., 2013; Mockdece Neves et al., 2016; Hernández-Martínez et al., 2017; Kagawa et al., 2023; Salas-Morillas et al., 2023) to estimate %BF, while two (7.4%) utilized DXA (Eufrásio de Medeiros et al., 2021; Remmel et al., 2021), and one (3.7%) used dual-frequency BIA (DF-BIA) (Kagawa et al., 2023). A limited number of studies also assessed FFM (Kagawa et al., 2023), lean body mass (Remmel et al., 2021), or fat-free mass index (FFMI) (Hernández-Martínez et al., 2017; Kagawa et al., 2023) via DF-BIA, DXA, and skinfold or DF-BIA techniques, respectively.

### 3.5 Body image assessment

Most studies assessed a component of negative body image (*n* = 22; 81.5%), while some (*n* = 7; 25.9%) analyzed the relationship between anthropometric and/or body composition indicators and aspects of positive body image. Among the 22 studies assessing negative body image, the Body Shape Questionnaire (BSQ) (Cooper et al., 1987) (*n* = 9; 40.9%) and the Stunkard FRS (Stunkard et al., 1983) (*n* = 5; 22.7%) were the most frequently administered, while three studies (13.6%) used the Contour Drawing Rating Scale (CDRS), developed by Thompson and Gray (Thompson and Gray, 1995). To assess aspects of positive body image, two studies (28.6%) each used the Body Appreciation Scale-2 (BAS-2) (Tylka and Wood-Barcalow, 2015a), Body Parts Shape and Size Scale-Revised (BPSS-R) (Petrie et al., 2002), Multidimensional Body-Self Relation Appearance Evaluation scale (MBSRQ-AS) (Cash, 2000), and Body Esteem Scale (Mendelson et al., 2001). The Feelings and Attitudes towards Body subscale of the Body Investment Scale (Orbach and Mikulincer, 1998) was also used in one study (14.3%) to assess an aspect of positive body image.

### 3.6 Body measures and aspects of negative body image

Higher BMI or %BF was significantly positively associated with an aspect of negative body image (e.g., body dissatisfaction, negative body attitudes) in 16 of the 22 studies (72.7%) (Table 3). Ten (62.5%) of these 16 studies assessed the relationship between anthropometry/body composition indicators and body image with bivariate Pearson or Spearman correlation, while the remaining studies (*n* = 6; 37.5%) used a combination of multivariate regression (e.g., stepwise, logistic), analysis of covariance, two sample, and path analyses. Significant positive correlation coefficients ranged from 0.170 to 0.657.

**Table 3:** Characteristics of studies that met the inclusion criteria and assessed an aspect of negative body image.

Author, (Year); Country	Sport(s)	*N* (% Female)	Age (SD)	Anthro/BC assessment method(s)	Anthro/BC indicator(s)	BI assessment tool(s)	BI outcome(s)	Main findings	Risk of bias
Aleksic-Veljkovic and Djurovic (2020); Serbia	Various sports	54 (100%)	19.0 (3.0)	Self-report questionnaire to inform height and weight	BMI	BSQ (Cooper et al., 1987); FRS (Stunkard et al., 1983)	BD; BD-S	BMI was positively correlated with BD and BD-S, in lean (BD: *r* = 0.342, *p* < 0.01; BD-S: *r* = 0.287, *p* < 0.05) and non-lean sport (BD: *r* = 0.371, *p* < 0.01; BD-S: *r* = 0.263, *p* < 0.05) athletes. BMI was positively correlated with BSQ in non-lean sport athletes (*r* = 0.267, *p* < 0.05), but not lean sport athletes (*r* = 0.106, *p* > 0.05).	Moderate
Cardoso et al. (2021); Brazil	Ballroom dancing	320 (42%)	31.5 (8.6)	Self-report questionnaire to inform height and weight	BMI	FRS (Stunkard et al., 1983), using Portuguese version (Scagliusi et al., 2006)	BD	The odds of being dissatisfied with the body due to excess weight were 51% greater in dancers with higher BMI compared to those with lower BMI (OR (95% CI): 1.518 (1.281–1.799), *p* < 0.001).	Low
Eufrásio de Medeiros et al. (2021); Brazil	Ballet	25 (100%)	24.0 (20.5–29.0)*	Height; weight; Lunar DXA	BMI; %BF	BSQ (Cooper et al., 1987), using Portuguese version (Di Pietro and Silveira, 2009); FRS (Stunkard et al., 1983), using version for Brazilian adults and children (Kakeshita et al., 2009)	BD	BMI (*r* = 0.657, *p* = 0.002) and %BF (*r* = 0.574, *p* = 0.010) were positively correlated with body dissatisfaction.	Low
de Sousa Fortes et al. (2015a); Brazil	Various sports	321 (0%)	17.3 (5.5)	Skinfolds (triceps, subscapular (all athletes), chest (≥18 years old))	%BF	BSQ (Cooper et al., 1987), using Portuguese version (Di Pietro and Silveira, 2009)	Body fat dissatisfaction	%BF and body fat dissatisfaction were positively correlated (*r* = 0.24, *p* < 0.01).	Low
de Sousa Fortes et al. (2015b); Brazil	Track & field	83 (100%)	15.1 (1.8)	Skinfolds (triceps. subscapular)	%BF	BSQ (Cooper et al., 1987), using version for Brazilian adolescents (Conti et al., 2009)	BD	%BF was significantly related to body dissatisfaction (*F*(1,82) = 28.50, *p* = 0.001).	Low
de Sousa Fortes et al. (2020); Brazil	Various sports	484 (100%)	17.1 (1.6)	Height; weight; skinfolds (triceps, subscapular)	BMI; %BF	BSQ (Cooper et al., 1987), using Portuguese version (Di Pietro and Silveira, 2009)	BD	BMI and %BF were positively correlated with body dissatisfaction (*r* = 0.20, *p* < 0.05).	Low
de Sousa Fortes et al. (2013); Brazil	Various sports	580 (20%)	10–19**	Height; weight; skinfolds (triceps, subscapular)	BMI; %BF	BSQ (Cooper et al., 1987), using version for Brazilian adolescents (Conti et al., 2009)	BD	%BF explained 18% of variance in BD in females and 13% in males. BMI explained 14% of variance, while BMI and %BF together explained 17% of variance in BD in males. All *p* < 0.01. The odds of BD among females with high %BF were 3.44 times the odds of BD if average %BF (95% CI: 1.29–9.17, *p* < 0.05). The odds of BD among males with obesity were 7.67 times odds of BD among males with normal weight (95% CI: 3.11–18.90, *p* < 0.05). The odds of BD among males with overweight was 5.85 times the odds of BD among males with normal weight (95% CI: 2.86–11.94; *p* < 0.05).	Low
Francisco et al. (2013); Portugal	Gymnastics, dance	131 (77%)	15.3 (2.2)	Unclear	BMI	CDRS (Thompson and Gray, 1995), using Portuguese version (Francisco et al., 2012)	BD	BMI was negatively correlated with BD in elite athletes (*r* = −0.42, *p* < 0.01).	Moderate
Godoy-Izquierdo and Díaz (2021); Spain	Soccer	45 (100%)	20.9 (7.5)	Self-report questionnaire to inform height and weight	BMI	FRS (Stunkard et al., 1983), using Spanish version (Ramírez et al., 2015, 2018); BAQ (Ben-Tovim and Walker, 1991), using Spanish version	Negative attitudes towards the body and appearance	BMI was positively correlated with negative body attitudes (BAQ: *r* = 0.44, *p* < 0.01).	Low
Goltz et al. (2013); Brazil	Various sports	156 (0%)	Weight class: 26.0 (7.1) Leanness: 31.7 (10.8) Aesthetic: 25.8 (8.9)	Height; weight; skinfolds (triceps, chest, mid-axillary, subscapular, suprailiac abdominal, medial thigh)	BMI; %BF	BSQ (Cooper et al., 1987), using Portuguese version (Di Pietro and Silveira, 2009)	BD	Athletes dissatisfied with their bodies (12.5 ± 5.9%) have a higher %BF than athletes satisfied with their bodies (9.7 ± 3.9%), *p* = 0.0034.	Moderate
Gomes et al. (2011); Portugal	Various sports	290 (48%)	17.8 (3.5)	Self-report questionnaire to inform height and weight	BMI	EDE-Q shape concern and weight concern subscales (Fairburn, 2008), using Portuguese version (Machado et al., 2014)	Shape concern, weight concern	BMI was positively correlated with shape (*r* = 0.17, *p* < 0.01) and weight concern (*r* = 0.25, *p* < 0.001)	Moderate
Hernández-Martínez et al. (2017); Spain	Weightlifting	32 (0%)	23.2 (8.0)	Height; weight; skinfolds (triceps, chest, subscapular, supraspinal, abdominal, anterior thigh); limb circumference	BMI; %BF; FFMI	ESM (González-Martí et al., 2012)	Muscle (dis)satisfaction	FFMI was highest among those with the greatest level of dissatisfaction with muscle (22.80 kg/m^2^) compared to FFMI of those with the middle (22.38 kg/m^2^) and lowest (22.30 kg/m^2^) levels of muscle dissatisfaction.	Moderate
Jardim et al. (2022); Brazil	Rhythmic gymnastics	18 (100%)	16.4 (4.0)	Self-report questionnaire to inform height and weight	BMI	BSQ (Cooper et al., 1987), using Brazilian version (Cordás and Castilho, 1994)	BD	BMI was positively correlated with body dissatisfaction (*r* = 0.51, *p* = 0.025).	Moderate
Kagawa et al. (2023); Japan	Long-distance running	31 (100%)	19.0 (18.0–20.0)*	Height; weight; skinfolds (triceps, subscapular, biceps, iliac crest, supraspinal, abdominal, front thigh, medial calf); girths (circumferences); lengths; breadths; DF-BIA	BMI; %BF; FM; FFM; FMI; FFMI; % abdominal fat; sum of 8 skinfolds	BAQ (Ben-Tovim and Walker, 1991), using Japanese version (Kagawa et al., 2007); BSS (Slade et al., 1990), using Japanese version (Ishigaki et al., 2009)	Negative attitudes towards body; BD	Biceps (*β* = 0.653, *p* = 0.002) and triceps (β = 0.610, *p* = 0.008) skinfolds and calf maximum girth (β = −0.625, *p* = 0.002) were associated with BAQ_Total._ BMI or body composition measurements were not associated with negative body attitudes or body dissatisfaction.	Low
Karr et al. (2013); USA	Various sports	627 (100%)	15.9 (1.2)	Self-report questionnaire to inform height and weight	BMI	EDI-3 BD subscale (Garner, 2004)	BD	BMI was positively associated with body dissatisfaction across all sport types (βs: 0.34–0.52, all *p* < 0.05)	Low
Mockdece Neves et al. (2016); Brazil	Artistic gymnastics	40 (93%)	13.3 (1.9)	Height; weight; skinfolds (triceps, subscapular)	BMI; %BF	BSQ (Cooper et al., 1987), using Spanish version (Conti et al., 2009)	BD	BMI and %BF were not significantly correlated with BSQ (BMI and BSQ: *r* = 0.262; %BF and BSQ: *r* = 0.159, all *p* > 0.05) and did not significantly explain body dissatisfaction among elite athletes.	Low
Pan et al. (2018); Taiwan and USA	Not defined	139 (41%)	19.4 (4.3)	Height; weight	BMI (categorical)	FRS (Stunkard et al., 1983)	BD	Athletes with overweight/obesity (M (SD): 2.53 (0.24)) had greater levels of BD compared to athletes without overweight/obesity (M (SD): 0.68 (0.19)). *F* (1, 128) = 36.86.	Low
Remmel et al. (2021); Estonia	Rhythmic gymnastics	33 (100%)	16.0 (12)	Height; weight; DXA	BMI; FM; LBM; %BF	BAT (Probst et al., 1995); subscale of factor 1: negative appreciation of body; subscale factor 3: general BD	Negative body attitudes	There were no significant relationships between BMI (*r* = 0.220), %BF (*r* = 0.040), FM (*r* = 0.065), nor LBM (*r* = 0.222) and BAT scores. All *p* < 0.05.	Moderate
Salas-Morillas et al. (2023); Spain	Acrobatic gymnastics	130 (87%)	14.1 (3.3)	Height; weight; skinfolds (triceps, biceps, subscapular, suprailiac, abdominal, medial calf)	BMI; Sum of six skinfolds; %BF	EDI-BD subscale (Garner, 2004)	BD	No anthropometric or body composition indicators were significantly correlated with BD (BMI: *r* = 0.162, *p* = 0.06; Sum of skinfolds: *r* = 0.094, *p* = 0.290; %BF_S_: 0.113, *p* = 0.202; %BF_Y_: *r* = 0.144, *p* = 0.1010).	Moderate
Voelker et al. (2018); USA	Figure skating	29 (0%)	18.5 (4.2)	Self-report questionnaire to inform height and weight	BMI	CDRS (Thompson and Gray, 1995)	BD-S	BMI was positively correlated with sport-related body dissatisfaction (*r* = 0.64, *p* < 0.01)	Moderate
Voelker et al. (2014); USA	Figure skating	272 (100%)	15.6 (3.0)	Self-report questionnaire to inform height and weight	BMI	CDRS (Thompson and Gray, 1995)	BD; BD-S; body image distortion	BMI was positively correlated with general BD (*r* = 0.63, *p* < 0.01) and sport-related BD (*r* = 0.62, *p* < 0.01).	Moderate
Whitehead et al. (2020); Australia	Physique sports: Bikini, fitness, figure, bodybuilding	348 (100%)	31.4 (8.1)	Self-report questionnaire to inform height and weight	BMI	EDI-BD subscale (Garner, 2004)	BD	BMI was positively correlated with body dissatisfaction (*r* = 0.279, *p* < 0.01).	Moderate

*Median (Q1-Q3); **Range. Anthro, Anthropometric; BC, Body Composition; BI, Body Image; BMI, Body Mass Index; %BF, Percent Body Fat; FFMI, Fat-Free Mass Index; FM, Fat Mass; FFM, Fat-Free Mass; LBM, Lean Body Mass; %FM, Percent Fat Mass; DF-BIA, dual-frequency-bioelectrical impedance analysis; BSQ, Body Shape Questionnaire; FRS, Figure Rating Scale; CDRS, Contour Drawing Rating Scale; BAQ, Body Attitudes Questionnaire; EDE-Q, Eating Disorder Examination-Questionnaire; ESM, Escala de Satisfacción Muscular; EDI-3, Eating Disorder Inventory-3; BAT, Body Attitude Test; BD, Body Dissatisfaction; BD-S, Sport-related Body Dissatisfaction; M, Mean; SD, Standard Deviation; OR, Odds Ratio; 95% CI, 95% Confidence Interval.

Similar findings were reported for sport-related body dissatisfaction. BMI showed a significant positive association with sport-related body dissatisfaction in all three (100%) studies that assessed this outcome (Voelker et al., 2014, 2018; Aleksic-Veljkovic and Djurovic, 2020).

#### 3.6.1 Age

Adolescent-only (12–19 years) participants were included in six (27.3%) of the 22 studies (Francisco et al., 2013; Karr et al., 2013; de Sousa Fortes et al., 2015b; Mockdece Neves et al., 2016; Remmel et al., 2021; Jardim et al., 2022). Among these studies, four (66.7%) reported significant associations between BMI and/or %BF and aspects of negative body image (Francisco et al., 2013; Karr et al., 2013; de Sousa Fortes et al., 2015b; Jardim et al., 2022). All three (100%) studies that included entirely adult populations found higher BMI and/or %BF was associated with greater body dissatisfaction (Whitehead et al., 2020; Cardoso et al., 2021; Eufrásio de Medeiros et al., 2021). The remaining thirteen (59.1%) studies included participants of heterogenous ages or did not provide information to determine age classification, and therefore, cannot contribute to this subanalysis.

#### 3.6.2 Gender

Eleven of the 22 studies (50.0%) assessing an aspect of negative body image had all-female participants (Karr et al., 2013; Voelker et al., 2014; de Sousa Fortes et al., 2015b, 2020; Aleksic-Veljkovic and Djurovic, 2020; Whitehead et al., 2020; Eufrásio de Medeiros et al., 2021; Godoy-Izquierdo and Díaz, 2021; Remmel et al., 2021; Jardim et al., 2022; Kagawa et al., 2023), while four (18.2%) included only males (Goltz et al., 2013; de Sousa Fortes et al., 2015a; Hernández-Martínez et al., 2017; Voelker et al., 2018). All but one (Remmel et al., 2021) of these studies reported significant positive associations between BMI, skinfolds or %BF and an aspect of body image disturbance. Among studies with only male participants, all four found positive relationships between BMI (Voelker et al., 2018), %BF (Goltz et al., 2013; de Sousa Fortes et al., 2015a), or FFMI (Hernández-Martínez et al., 2017) with general (Goltz et al., 2013; de Sousa Fortes et al., 2015a), sport-related (Voelker et al., 2018), or muscle dissatisfaction (Hernández-Martínez et al., 2017) (Table 3). The remaining studies (*n* = 7) included a combination of female and male athletes, and the results were mixed among these studies as four (57.1%) reported significant positive associations between anthropometric and/or body composition indicators, one (14.3%) reported a significant negative association, and two (28.6) reported no significant associations.

#### 3.6.3 Sport type

Twelve studies (54.5%) assessing a component of negative body image included only lean sport athletes. Among these studies, two-thirds (*n* = 8; 66.7%) found BMI and/or %BF was positively correlated with an aspect of negative body image, while an inverse (Francisco et al., 2013) or no significant relationship (Mockdece Neves et al., 2016; Remmel et al., 2021; Salas-Morillas et al., 2023) was reported in the remaining four (33.3%) studies. In the two (9.1%) studies that assessed body dissatisfaction in only non-lean sport athletes, significant positive findings were reported with BMI (Godoy-Izquierdo and Díaz, 2021) and FFMI (Hernández-Martínez et al., 2017). Two (9.1%) studies included a mixture of both lean and non-lean sport athletes (Karr et al., 2013; Aleksic-Veljkovic and Djurovic, 2020); BMI was positively associated with at least one measurement of body image dissatisfaction in all athlete groups.

#### 3.6.4 Body measures

All ten studies (100%) that used self-reported anthropometric data found significant associations between BMI and an aspect of negative body image, measured by questionnaires (e.g., BSQ, BAQ) and rating scales (e.g., FRS, CDRS). A majority of studies (*n* = 7; 58.3%) that used researcher-measured anthropometric and/or body composition indicators reported significant findings.

Eleven studies (50%) assessed %BF or FM (de Sousa Fortes et al., 2013, 2015a,b, 2020; Goltz et al., 2013; Mockdece Neves et al., 2016; Hernández-Martínez et al., 2017; Eufrásio de Medeiros et al., 2021; Remmel et al., 2021; Kagawa et al., 2023; Salas-Morillas et al., 2023), using skinfolds (de Sousa Fortes et al., 2013, 2015a,b, 2020; Goltz et al., 2013; Mockdece Neves et al., 2016; Hernández-Martínez et al., 2017; Salas-Morillas et al., 2023), DXA (Eufrásio de Medeiros et al., 2021; Remmel et al., 2021), or bioimpedance techniques (Kagawa et al., 2023), and ten (90.9%) reported the results of quantitative assessments with an aspect of negative body image. Six of the 10 studies (60%) reported body fat was positively associated with body dissatisfaction and/or negative body attitudes (de Sousa Fortes et al., 2013, 2015a,b, 2020; Goltz et al., 2013; Eufrásio de Medeiros et al., 2021) in female (de Sousa Fortes et al., 2015b, 2020; Eufrásio de Medeiros et al., 2021), male (Goltz et al., 2013; de Sousa Fortes et al., 2015a), and combined populations (de Sousa Fortes et al., 2013). Of these studies, most used skinfolds to generate estimates of %BF (de Sousa Fortes et al., 2013, 2015a,b, 2020; Goltz et al., 2013), while one (10%) utilized DXA (Eufrásio de Medeiros et al., 2021). A smaller number of studies (*n* = 3; 13.6%) assessed FFM or lean body mass, or FFMI (Hernández-Martínez et al., 2017; Remmel et al., 2021; Kagawa et al., 2023). Only one (33.3%) of these three reported a significant association with an aspect of negative body image. (Hernández-Martínez et al., 2017). Two studies (66.7%) reported non-significant associations between FFM and lean body mass (Remmel et al., 2021; Kagawa et al., 2023). Notably, one of these studies also conducted a comprehensive anthropometric profile for participants, and found mixed associations of the various anthropometric indicators with negative body image attitudes (Kagawa et al., 2023).

### 3.7 Body measures and aspects of positive body image

Findings among the seven studies (25.9%) assessing an aspect of more positive body image (Petrie et al., 2014; Galli et al., 2015; Kantanista et al., 2018; Kosmidou et al., 2018; Soulliard et al., 2019; Godoy-Izquierdo and Díaz, 2021; Jardim et al., 2022) were less consistent (Table 4). Three studies reported no associations between BMI and an aspect of positive body image (Galli et al., 2015; Soulliard et al., 2019; Jardim et al., 2022), while the remaining four showed that BMI was significantly inversely correlated with or predictive of at least one aspect of positive body image (Petrie et al., 2014; Kantanista et al., 2018; Kosmidou et al., 2018; Godoy-Izquierdo and Díaz, 2021). Of two studies assessing body appreciation, a true component of positive body image, one reported a negative association with BMI (Godoy-Izquierdo and Díaz, 2021), while the other found BMI did not significantly predict body appreciation among athletes (Soulliard et al., 2019). Two studies used BPSS-R and MBSRQ-AS to assess body shape and size satisfaction and overall appearance satisfaction. One found that increased BMI was associated with decreased body satisfaction (Petrie et al., 2014), while the other did not (Galli et al., 2015). Two studies assessed body esteem and both reported negative, but non-significant correlations with BMI (Kosmidou et al., 2018; Jardim et al., 2022). However, one of these studies additionally used hierarchical regression and found BMI was a significant predictor of lower body esteem (Kosmidou et al., 2018), similar to an additional regression analysis which reported BMI as a significant predictor of lower positive body image scores (Kantanista et al., 2018).

**Table 4:** Characteristics of studies that met the inclusion criteria and assessed an aspect of positive body image.

Author (Year); Country	Sport(s)	*N* (% Female)	Age (SD)	Anthro/BC assessment method(s)	Anthro/BC indicator(s)	BI assessment tool(s)	BI outcome(s)	Main findings	Risk of bias
Galli et al. (2015); USA	Various sports	183 (0%)	20.3 (1.7)	Self-report questionnaire to inform height and weight	BMI	BPSS-R (Petrie et al., 2002); MBSRQ-AS (Cash, 2000)	Body shape and size satisfaction; overall appearance satisfaction	BMI was not correlated with body satisfaction (BPSS: *r* = 0.04; MBSRQ-AS: *r* = −0.06; *p* > 0.05).	Moderate
Godoy-Izquierdo and Díaz (2021); Spain	Soccer	45 (100%)	20.9 (7.5)	Self-report questionnaire to inform height and weight	BMI	BAS-2 (Tylka and Wood-Barcalow, 2015a), using Spanish version (Swami et al., 2017)	Body appreciation	BMI was inversely correlated with body appreciation (*r* = −0.37, *p* < 0.05).	Low
Jardim et al. (2022); Brazil	Rhythmic gymnastics	18 (100%)	16.4 (4.0)	Self-report questionnaire to inform height and weight	BMI	BES (Mendelson et al., 2001), using Brazilian version (Caetano, 2011)	Body esteem	BMI was not correlated with body esteem (*r*^2^ = − 0.403, *p* = 0.087).	Moderate
Kantanista et al. (2018); Poland	Various sports	242 (100%)	20.0 (4.5)	Self-report questionnaire to inform height and weight	BMI	Feelings and Attitudes towards Body Scale (part of Body Investment Scale) (Orbach and Mikulincer, 1998), using Polish version (Nalecz et al., 2012)	BI	BMI was inversely associated with body image and explained 3.6% of variance in body image (*R*^2^ = 0.036, *β* = −0.230, *p* < 0.001).	Moderate
Kosmidou et al. (2018); Greece	Rhythmic gymnastics	49 (100%)	12.4 (1.7)	Self-report questionnaire to inform height and weight	BMI	BES (Mendelson et al., 2001), using Greek version	Body esteem	BMI was a significant predictor of body esteem (*β* = −0.09, *p* = 0.01) with hierarchical regression analysis, but BMI was not correlated with body esteem (*r* = −0.17, *p* > 0.05).	Moderate
Petrie et al. (2014); USA	Various sports	203 (0%)	20.3 (1.6)	Self-report questionnaire to inform height and weight	BMI	BPSS-R (Petrie et al., 2002); MBSRQ-AS (Cash, 2000)	Body shape and size satisfaction; overall appearance satisfaction	BMI was inversely correlated with body satisfaction (BPSS: *r* = −0.22, *p* < 0.001; MBSRQ-AS: *r* = −0.18, *p* < 0.05).	Low
Soulliard et al. (2019); USA	Various sports	79 (67%)	19.8 (1.1)	Self-report questionnaire to inform height and weight	BMI	BAS-2 (Tylka and Wood-Barcalow, 2015a)	Body appreciation	BMI did not significantly predict body appreciation among athletes (β = −0.06, *t* = 0.49, *p* = 0.62, *R*^2^ < 0.01).	Low

Anthro, Anthropometric; BC, Body Composition; BI, Body Image; BPSS-R, The Body Parts Satisfaction Scale-Revised; MBSRQ-AS; The Multidimensional Body-Self Relations Questionnaire-Appearance Scale; FRS, Figure Rating Scale; BAS-2, The Body Appreciation Scale-2; The BES, Body Esteem Scale; BMI, Body Mass Index; PBI, Perceived Body Image; IBI, Ideal Body Image; SD, Standard Deviation.

#### 3.7.1 Age

All but one study (Jardim et al., 2022) (*n* = 6; 85.7%) assessing an aspect of positive body image included participants that crossed multiple age categories. Three (42.9%) assessed the relationship between BMI and body shape and size satisfaction and overall appearance satisfaction (Petrie et al., 2014; Galli et al., 2015), or body appreciation (Soulliard et al., 2019) in collegiate athletes. Two (Petrie et al., 2014; Galli et al., 2015) of the three (66.7%) collegiate population analyses (Petrie et al., 2014; Galli et al., 2015; Soulliard et al., 2019) did not provide minimum and maximum age ranges, so classifying the study populations as adults was not possible. Nevertheless, one of the three (33.3%) collegiate athlete analyses reported BMI was inversely correlated with body satisfaction (Petrie et al., 2014), while the other two (66.7%) did not find significant associations between BMI and body satisfaction (Galli et al., 2015) and body appreciation (Soulliard et al., 2019). In one study of only adolescents (12–19 years), BMI and body esteem were not significantly associated (Jardim et al., 2022). The remaining studies (*n* = 3; 42.9%) had unspecified (Kosmidou et al., 2018) or broad age ranges (Kantanista et al., 2018; Godoy-Izquierdo and Díaz, 2021).

#### 3.7.2 Gender

Four studies (57.1%) assessed aspects of positive body image in all-female populations and all reported inverse associations with BMI (Kantanista et al., 2018; Kosmidou et al., 2018; Godoy-Izquierdo and Díaz, 2021; Jardim et al., 2022). Two studies assessing body appreciation, a primary aspect of positive body image, found inverse correlations between BMI and body appreciation, measured with the BAS-2 (Godoy-Izquierdo and Díaz, 2021). Results were inconsistent between the two studies that analyzed the relationship between BMI and body shape and size satisfaction (BPSS-R) and overall appearance satisfaction (MBSRQ-AS) in US male athletes of various sports (Petrie et al., 2014; Galli et al., 2015). One reported significant inverse correlations between BMI and body and overall appearance satisfaction (Petrie et al., 2014), while the other reported non-significant findings (Galli et al., 2015). The final study that assessed an aspect of positive body image included women and men and found BMI did not significantly predict body appreciation (Soulliard et al., 2019), when analyzing all athletes together.

#### 3.7.3 Sport type

Among the seven studies assessing an aspect of positive body image only three (42.9%) could be categorized as having entirely lean (Kosmidou et al., 2018; Jardim et al., 2022) or non-lean (Godoy-Izquierdo and Díaz, 2021) athlete populations. Mixed results were reported among the two studies that assessed body esteem in lean sport athletes (Kosmidou et al., 2018; Jardim et al., 2022), while the one study of non-lean sport athletes found a significant inverse correlation between BMI and body appreciation (Godoy-Izquierdo and Díaz, 2021). The remaining four studies (57.1%) included a combination of lean and non-lean sport athletes.

#### 3.7.4 Body measures

All studies (*n* = 7; 100%) assessing an aspect of positive image used self-report questionnaires to obtain participant heights and weights to calculate estimates of BMI. Therefore, there is no available data to explore potential variations in the relationship between body composition and components of positive body image based on the method of body composition assessment.

## 4 Discussion

This review systematically evaluated the association between anthropometric and/or body composition indicators and body image in athletes. Twenty-seven cross-sectional studies met the criteria for inclusion, such that the participants were athletes (see prior definition of athlete) in whom the relationship between anthropometric and/or body composition indicators and body image was quantitatively assessed. We explored whether the relationship between body measures and body image differed according to the body image outcome assessed (i.e., aspect of negative vs. positive body image). Within each outcome categorization, the potential role of age, gender, the method of anthropometric or body composition assessment, and sport type were also considered. All included studies were of moderate or high quality.

These studies generally indicated positive associations between body measures and aspects of negative body image (e.g., body dissatisfaction, negative body attitudes), such that higher BMI and/or %BF was associated with greater body image disturbance. There were negative, but less consistent, associations between BMI and aspects of positive body image. These observed relationships were generally consistent across sexes, age groups, sport type (i.e., lean vs. non lean), and methods of assessing body metrics (i.e., self-report vs. researcher measured), yet there were a few possible deviations.

Although body dissatisfaction is more prevalent in lean-sport athletes (Kong and Harris, 2015; Chapa et al., 2022), a relatively lower proportion of studies detected significant associations between body measures and an aspect of negative body image in lean sport athletes compared to non-lean sport athletes. This may be explained in part by potential differences in the BMIs of athletes from lean sports compared to those of non-lean sport athletes. For example, the mean BMI of athletes from studies including only lean sports and finding non-positive associations ranged from 18.2 to 20.4 kg/m^2^, while the one study that assessed BMI in non-lean sports, reported a mean BMI of 23.1 kg/m^2^, with a range of 16.2–33.6 kg/m^2^. Therefore, although previous findings that suggest “protective” effects of sport participation on body image concerns may be mitigated in sports that encourage thinness and leanness for aesthetic standards or performance (Varnes et al., 2013; Chapa et al., 2022), we may have observed fewer associations between body size and composition in lean sport athletes due to lower body measures. Additionally, our findings may be due to our dichotomous classification of athletes as lean/non-lean sport athletes, which may have missed the nuances of aesthetic/lean vs. non-aesthetic/lean sports. One major challenge in assessing the effect of sport type is the varied categorizations of sport across studies and reviews. For example, classifications can be made based on aesthetic vs. nonaesthetic sports (Zaccagni and Gualdi-Russo, 2023), while others consider more granular categorizations, comparing lean, feminine, and aesthetic sport athletes (Varnes et al., 2013) or aesthetic, endurance, and ball game athletes (Hausenblas and Symons Downs, 2001).

Notably, all studies that used self-report questionnaires to obtain participant BMI reported significant associations between these metrics and an aspect of negative body image, while a smaller percentage (*n* = 7; 58.3%) of studies using researcher-measured anthropometric or body composition outcomes found similar significant positive associations. Self-report questionnaires are often an appealing method of obtaining participant height and weight in research settings due to limited participant and researcher burden. In vulnerable populations of adolescents and athletes, these questionnaires may have additional advantages. Weighing athletes may not be ideal for this population, as team weigh-ins have been associated with increased dietary restriction (Galli et al., 2017). Many of the included studies that used researcher-measured methods, however, used approaches to eliminate these concerns like having participants complete body image questionnaires and scales prior to undergoing body measures and completing body measures in a private setting (i.e., away from teammates and coaches). Despite the perceived advantages of self-reported height and weight questionnaires, BMI itself has potential limitations. While BMI is a surrogate measure of body fat that may be an acceptable indicator of body fatness for some, it does not differentiate between body components (i.e., FM vs. FFM) and cannot determine fat distribution (Kruschitz et al., 2013). These characteristics may further limit its utility in athlete populations with larger percentages of muscle mass. Given the known limitations of BMI, particularly among athletic populations, it is critical that future work continues to prioritize the application of techniques that can generate indicators of body composition.

Previous systematic reviews and meta-analyses have focused on differences in body image between athletes and non-athletes, generally finding that participation in athletics may confer some protection against body image concerns (Hausenblas and Symons Downs, 2001; Varnes et al., 2013; Chapa et al., 2022; Burgon et al., 2023). For example, Hausenblas and Symons Downs reported less body dissatisfaction in male and female athletes compared to nonathletes (Hausenblas and Symons Downs, 2001). In an all-female sample, Varnes et al. found that female collegiate athletes had fewer body image concerns and desire to look athletic and obtain a more muscular physique compared to non-athletes (Varnes et al., 2013). Chapa et al. and Burgon et al. also reported lower levels of body image concerns in athletes compared to non-athletes, among female athletes and athletes of both sexes, respectively (Chapa et al., 2022; Burgon et al., 2023).

This review is novel in its focus on the relationship of anthropometrics and/or body composition indicators with body image in athletes. Only two previous reviews have reported on associations of body measures and body image among athletes, both doing so as secondary objectives (Hausenblas and Symons Downs, 2001; Zaccagni and Gualdi-Russo, 2023). In a 2001 review, Hausenblas and Symons Downs did not find differences in the magnitude of the effect size between individuals with BMI < 20 kg/m^2^, 20–22 kg/m^2^, and > 22 kg/m^2^ (Hausenblas and Symons Downs, 2001). However, a more recent review by Zaccagni and Gualdi-Russo suggested that underweight athletes were more dissatisfied with their bodies than those of a normal weight (Zaccagni and Gualdi-Russo, 2023). Importantly, this result was generated from a meta-analysis of only one study, consisting of 81 athletes (Borrione et al., 2013). This meta-analysis was possible because descriptive statistics were provided separately for athletes of different competition levels, who were therefore treated as distinct populations (Borrione et al., 2013; Zaccagni and Gualdi-Russo, 2023). Crucially, this analysis relied on the assumption that the average BMI for each group of competitors could be uniformly applied to all group members.

Our findings add to the sparse prior analyses and demonstrate that athletes’ weight status and body composition are associated with body dissatisfaction, similar to the general population. Sport participation can impact how athletes appreciate their bodies and perceive them with regard to their utility and importance to their athletic success (Wiggins and Moode, 2000; Soulliard et al., 2019). However, athletes are believed to have multiple body images that vary according to context, such that they possess an athletic body image in addition to a body image based on social ideals (Greenleaf, 2002; de Bruin et al., 2011). The findings of the present review suggest that athletes are still subject to the social ideals and norms that contribute to body image disturbance in the general population, as demonstrated previously (Russell, 2004; Karr, 2011).

This review was novel in its primary focus on body measures as a factor related to body image in athletes. A comprehensive search was completed in five databases with the guidance of a research librarian, and the input of respective body composition and disordered eating experts. The studies included in this review spanned many countries and continents, and we found consistent positive associations between BMI and/or %BF and aspects of negative body image, increasing the generalizability of these results. Given the influence of age, sex, and sport type on the indicators and outcomes assessed, this review importantly assessed associations between anthropometric and body composition indicators and body image in athletes across levels of these variables. Another strength of the present review was including outcomes related to aspects of negative and positive body image. In recent years, positive body image research has expanded, and it is important to understand whether the relationships between body measures and aspects of negative and positive body image are similar given that the constructs are unique.

This review has several important limitations. For inclusion in the present review, reports had to be available in the English language. Therefore, it is probable that relevant findings were excluded from this review based on language alone. The final 27 reports included in the review, however, represented studies conducted across 11 countries and five continents, indicating the broad geographical representation of the findings. Furthermore, the present review did not restrict the age range of participants. Body dissatisfaction is highly prevalent among adolescents, and longitudinal analyses have demonstrated increases in body dissatisfaction from adolescence through emerging adulthood (Bucchianeri et al., 2013; Quick et al., 2013). We aimed to be inclusive with regard to age as this is one of the first reviews of its kind in athletes. Across the studies included in the present review, we also identified challenges. First, the cross-sectional nature of all included studies limits or precludes causal inferences regarding the relationship between body measures and body image in athletes. Future studies utilizing longitudinal data will permit the assessment of temporality to provide important insights into the direction and timing of the association. Second, many studies failed to operationalize the term ‘athlete,’ and provide limited participant details, including the training and competition level (s). Third, most studies included in this review relied on self-report questionnaires to gather height and weight data for BMI calculations. Although measured and self-reported BMI correlate highly (Keith et al., 2011; Lipsky et al., 2019), adolescents and adults may systematically overestimate height and underestimate weight, leading to underestimations of BMI with self-report (Gorber et al., 2007; Sherry et al., 2007). This appears most problematic when attempting to categorize individuals according to weight status (Keith et al., 2011), rather than using continuous BMI, as did many of the included studies. Fourth, few studies evaluated body composition, instead, the majority used BMI as the measure of body size. Finally, among the studies that evaluated a body composition indicator, many utilized skinfold thickness measurements from a limited number of sites (i.e., 2–3 sites) to estimate an aspect of body composition. Although skinfold thickness correlates more strongly with %BF than BMI (Sarría et al., 1998; Wohlfahrt-Veje et al., 2014), the accuracy of estimations of %BF from skinfold measurements may vary depending on BMI (Freedman et al., 2007) and by the skinfold sites and equations utilized (Lohman et al., 1984).

Future research should aim to include more robust assessments of the relationship between body composition and body image in athletes. This would include longitudinal study designs to understand the temporality and stronger analytical methods that include relevant covariates, compared to the simpler correlation analyses used in the majority of the studies included in the present review. Additionally, future studies should continue assessing components of positive body image to provide insights into the potential differing associations between body measures and aspects of negative and positive body image. To better understand the nuances of the body size-body image relationship in athletes, forthcoming studies should utilize more accurate researcher-measured body composition techniques, rather than rely on self-reported height and weight. Overall, these considerations will deepen our understanding of body composition components as factors affecting athlete body image. These findings can inform future research towards athletes that may require additional attention and resources to achieve optimal body image.

## Data availability statement

The original contributions presented in the study are included in the article/Supplementary material, further inquiries can be directed to the corresponding author.

## Author contributions

MW: Conceptualization, Formal analysis, Investigation, Methodology, Writing – original draft, Writing – review & editing. MM: Formal analysis, Investigation, Methodology, Project administration, Writing – review & editing. CE: Conceptualization, Methodology, Writing – review & editing. SK: Methodology, Writing – review & editing. CP: Conceptualization, Investigation, Supervision, Writing – review & editing.
